# Precise acetabular positioning, discrepancy in leg length, and hip offset using a new seven-axis robot-assisted total hip arthroplasty system requires no learning curve: a retrospective study

**DOI:** 10.1186/s13018-023-03735-3

**Published:** 2023-03-24

**Authors:** Run Tian, Xudong Duan, Ning Kong, Kunzheng Wang, Pei Yang

**Affiliations:** Department of Bone and Joint Surgery, The Second Affiliated Hospital of Xi’an Jiaotong University, Xi’an, 710004 China

**Keywords:** Total hip arthroplasty, Learning curve, Robot-assisted system, Cumulative sum analysis, Cup positioning

## Abstract

**Objective:**

The purpose of the present study was to determine the learning curve for a novel seven-axis robot-assisted total hip arthroplasty (RA-THA) system, and to explore whether it was able to provide greater accuracy in acetabular cup positioning, superior leg length discrepancy (LLD), and hip offset than conventional methods.

**Methods:**

A total of 160 patients in which unilateral THA was performed in the second affiliated Hospital of Xi'an Jiaotong University from July 2021 to September 2022 were studied. The first 80 patients underwent robot-assisted THA, while conventional THA was performed on the subsequent 80 by the same team of experienced surgeons. The learning curve for the RA-THA system was evaluated using cumulative sum (CUSUM) analysis. The demographic data, preoperative clinical data, duration of surgery, postoperative Harris hip score (HHS) and postoperative radiographic data from patients that had conventional THA were compared.

**Results:**

The 80 patients who underwent primary unilateral RA-THA comprised 42 males and 38 females and were followed up for 12 weeks. Using analysis by CUSUM, the learning curve of the RA-THA system could be divided into learning and proficiency phases, the former of which consisted of the first 17 cases. There was no significant difference between the learning and proficiency phases in terms of LLD, hip offset, or accuracy of acetabular prosthesis position in the RA-THA groups. The proportion of acetabular prostheses located in the Lewinnek safe zone was 90.5% in the proficiency group and 77.5% in the conventional group, respectively, a difference that was statistically significant (*P* < 0.05). The absolute error between target angle and postoperative measured angle of anteversion was statistically significant in the proficiency group and the conventional group((*P* < 0.05). Postoperative acetabular anteversion and LLD were 19.96 ± 5.68° and 6.00 (5.00) mm in the proficiency group, respectively, and 17.84 ± 6.81° and 8.09 (4.33) mm using conventional surgery, respectively (anteversion: *P* = 0.049; LLD: *P* < 0.001).

**Conclusions:**

The surgical team required a learning curve of 17 cases using the RA-THA system to become proficient. There was no learning curve for other parameters, namely LLD, hip offset, or accuracy of acetabular prosthesis positioning. During the proficiency phase, the RA system was superior to conventional THA for control of leg length and accuracy of acetabular cup placement.

## Introduction

It is widely accepted that total hip arthroplasty (THA) is among the most effective orthopedic procedures for restoration of hip function. Current data indicate that more than one million people undergo THA each year worldwide, with more than 400,000 in China in 2018 [1, 2]. Although conventional THA is a mature procedure, it requires substantial surgical experience. It is difficult to perform accurate manual prosthesis positioning and implantation. Consequently, aseptic loosening, dislocation, leg length discrepancy (LLD), wear to the prosthesis surface, in addition to other problems, remain to be solved [3, 4]. This often leads to early revision surgery, affecting the quality of life for patients and also imposes an additional financial burden. Robot-assisted (RA) THA has been widely used clinically since it allows preoperative planning, fewer intraoperative mistakes, and greater accuracy regarding implantation of the prosthesis [5–7]. Previous studies have demonstrated that RA-THA can achieve superior acetabular component positioning to provide equal leg length, improving offset, and reducing postoperative complications and rates of early revision [6–9]. In addition, the literature predicts that RA systems will become more prevalent [10]. Nevertheless, as a new technology, RA-THA still requires surgeons to undergo a substantial degree of learning to optimize safety and reproducibility. The respective learning curve can describe the dynamic change in the surgeon's surgical proficiency using a particular system.

A number of studies have reported the learning curve characteristics of RA-THA systems, but mostly those developed in the USA and Europe [11, 12]. Few studies have evaluated the learning curves of such systems designed in China, and so their characteristics require further exploration. Therefore, the present retrospective study was conducted to review the outcomes of a novel seven-axis robot-assisted THA system (Jianjia, Hangzhou Jianjia Robot Co., Ltd.). The purpose of this study was: (i) to evaluate the learning curve of the RA-THA system, (ii) to confirm whether it could achieve superior clinical and radiographic results than conventional methods by analysis of the learning curve.

## Materials and methods

### Study design

Patients were included in the study based on the following inclusion criteria: (1) Garden type III or IV femoral neck fracture, Ficat stage III and IV femoral head necrosis, or hip osteoarthritis (Including hip dysplasia Crowe type I, type II); (2) Unilateral THA with normal shape and function of the contralateral hip joint; Exclusion criteria: (1) Local anatomical abnormalities caused by previous fractures or surgical history related to the proximal femur or acetabulum; (2) Intraoperative femoral osteotomy; (3) Deformities or abnormal development of the pelvis or lower extremities; (4) non-standard plain film of the pelvis or the apex of the greater trochanter or pelvic teardrop not clearly visible on plain film.

Non-excluded patients satisfying the inclusion criteria that underwent unilateral THA at the second affiliated Hospital of Xi'an Jiaotong University from July 2021 to September 2022 were included, representing the first 80 patients who received RA-THA and 80 that received conventional THA. Meanwhile, The primary diseases of all patients were also recorded. The retrospective study was approved by the Ethics Committee of the second affiliated Hospital of Xi'an Jiaotong University (Permit Number: 2020–935).

### Preoperative preparations

In the RA-THA group, the patients underwent preoperative CT scanning of the bilateral hip and knee joints. The data were uploaded into the Jianjia robotic system to construct 3D models for preoperative planning. The acetabular cup placement angle for all operations was set to an anteversion angle of 20.0° and an inclination angle of 40°(radiographic inclination of 42° and radiographic anteversion of 15°) relative to the functional pelvic plane (FPP). The surgeon was then able to choose an appropriate femoral prosthesis, in accordance with the preoperative plan, to improve bone contact and achieve the correct leg length and offset. The surgeon was also able to adjust the position of the acetabular cup to improve the range of motion and stability of the hip joint. In the conventional THA group, the acetabular cup placement angle for all operations was set to an anteversion angle of 20.0° and an inclination angle of 40°(radiographic inclination of 42° and radiographic anteversion of 15°) relative to the FPP, and patients were assessed preoperatively using standard pelvic plain film. During surgery, the acetabular cup position is determined by using mechanical navigation. Standard pelvis X-rays were taken as follows: the x-ray projection center was on the midpoint of bilateral hip, the projection distance was 100 cm, and legs were straight and internally rotated by 15 ~ 20 º. In actual measurements, the angle between the long axis of the femoral shaft and the long axis of the pelvis can be used as the criterion for judging whether the plain film is qualified. If the angle is greater than 10º, the plain film will be judged to be non-standard. Preoperative template measurement was conducted on plain film to select the type of prosthesis and plan the location of surgery.

### Surgical technique

All patients received combined general anesthesia. The THA procedure was performed using a posterolateral approach. The pelvic position was obtained using a signal matrix mounted 4 cm above the anterior superior iliac crest on the side to be restored. Following exposure of the acetabulum, posterior dislocation of the hip was performed and femoral osteotomy conducted 1 cm above the lesser trochanter. Gauze strips were used to fill the prepared medullary cavity. After exposure to the acetabulum, the hip joint was posteriorly dislocated and femur osteotomy was performed 1 cm above the lesser trochanter. Then the medullary cavity is prepared and filled with gaze strips. Afterward, the acetabulum was exposed and the labrum and part of the joint capsule were removed, and the acetabulum position was registered with a probe. At hip registration, a probe was used to determine the anterior and posterior notch of the fossa ovalis and the superior apex of the acetabulum. Then, according to the preoperative planning, a probe was used to select 25–35 points in the acetabulum to ensure that the probe penetrated through the cartilage and explored the bone surface. After all feature points are registered, if the bias is less than 0.1, the acetabulum is successfully registered. Once the above steps were completed, the acetabulum was rasped and filed with a mechanical arm under the limits of the defined inclination and anteversion. After filing, the soft tissue in the acetabulum was cleaned again, and the acetabular prosthesis was installed with the assistance of the mechanical arm, and the position of the prosthesis was verified. The femoral component was installed and its stability was confirmed after the reduction in the hip.

The same team of surgeons performed all surgery and placed each non-cemented joint prosthesis (BE: femoral prosthesis; Chunli: polyethylene components; 58 acetabular prosthesis; Chunli Company). The team had no previous experience of a robot-assisted system.

### Follow-up and radiographic measurement

Up to 7 days following surgery, radiographs of the anteroposterior pelvis were obtained from each patient to measure LLD and hip offset. Computed tomography (CT) scans of the hip were also performed to measure the inclination and anteversion angles of the cup. To reduce measurement bias, all data were measured independently by two trained radiologists in a randomized order, and the mean of the two measurements was recorded.

### Leg length discrepancy (LLD)

LLD was measured as the vertical distance between the bilateral acetabular teardrop line and the line connecting the most prominent point of each lesser trochanter on the bilateral femurs. Generally, an LLD > 10 mm was considered unacceptable [13, 14].

#### Hip offset

The hip offset was measured from the pubic symphysis and the long axis of the femoral shaft and used to evaluate the hip stability. The difference between bilateral offsets was the offset discrepancy (Offset-D). An Offset-D greater than 5 mm was considered unacceptable [14–16].

#### Inclination and anteversion angles

The Lewinnek safe zone was used to assess whether the position of the prosthesis was appropriate (inclination angle 30- 50°, anteversion angle 5- 25°) [17]. Any prostheses that exceeded this acceptable angle range were recorded as malposition of the acetabulum.

Inclination of the cup is represented by the angle between the long axis of acetabular cup and bilateral teardrop line, which was considered acceptable at 40 ± 10°.

Anteversion of the cup was measured from the residual angle formed between a line between the anterior and posterior edges of the acetabular prosthesis and a line between the center points of the bilateral hip joints. A measurement of 15 ± 10° was considered acceptable [14], [18].

The radiographic inclination (RI) and anatomical anteversion (AA) of the acetabular prosthesis were measured through CT. According to Murray's anteversion conversion formula [19], radiographic anteversion = ${\mathrm{tan}}^{-1}(\mathrm{tan}AA\times \mathrm{sin}RI)$, we calculated and recorded the radiographic anteversion of the acetabular prosthesis. Furthermore, to analyze the accuracy of the robot-assisted system, the absolute error between the target angles and postoperative measured angles was calculated.

### Harris hip score

Harris hip score (HHS) values were collected both prior to and 12 weeks post-operation. The HHS is widely used in the evaluation of hip function from multiple outcome measures, including pain, walking function, daily living activity, and the range of motion of the hip joint [20]. Any clinical complications identified during the study were documented.

### CUSUM analysis

CUSUM analysis is a statistical method used to evaluate a learning curve graphically and is considered a mature tool for quality control in healthcare [21]. It magnifies trends in data variation and identifies an inflection point by calculating ordinal differences between each sequential data value and the cumulative mean value. The cumulative sum was calculated as follows: CUSUM = ${\sum }_{i=1}^{n}({X}_{i} -\mathrm{ U})$, where ${X}_{i}$ represents the duration of surgery for each patient, $\mathrm{U}$ represents the mean duration for all cases, and n represents the sequence number of each operation. Surgery that was of greater duration increased the CUSUM value, while shorter surgery reduced the CUSUM value [14]. A learning curve was plotted with case number on the x-axis and each sequential CUSUM value was calculated from the difference of the duration of surgery from the overall mean, on the y-axis, then fitted to a polynomial equation. Fitting was considered successful when *P* < 0.05 and judged according to R [2]. With the inflection point of the curve representing the minimum number of surgical cases required to cross the learning curve threshold, the curve was divided into two different phases: the learning phase and proficiency phase. The curve was divided into two learning stages separated by the apex of the curve, representing the point at which the slope changed from positive to negative, and also considered the minimum cumulative number of operations required for a surgeon to cross the threshold of the learning curve.

### Statistical analysis

A Kolmogorov–Smirnov test was used to evaluate the normal distribution of the quantitative data, including patient demographic data, imaging parameters, and preoperative and postoperative scores. Normally distributed measurements are presented as means ± SD. Measurements with skewed distributions are presented as medians (interquartile range) [M(IQR)] and all categorical data as percentages. Levene's test was used to evaluate the homogeneity of data variance. The significance of normally distributed data was evaluated using a student's *t*-test. A Wilcoxon test was used for skewed distribution data, and a chi-square test for categorical data. The intraclass correlation coefficient (ICC) is used to review reliability in intra-observer and inter-observer: 0.81 to 1.00, nearly perfect reliability; 0.61 to 0.80, strong reliability; 0.41 to 0.60, moderate reliability; 0.21 to 0.40, fair reliability; and 0 to 0.20, poor reliability. Differences were considered statistically significant when *P* < 0.05. All statistical analyses were performed using SPSS software (version 25.0, SPSS, New York, NY, USA).

## Results

**Fig. 1:**
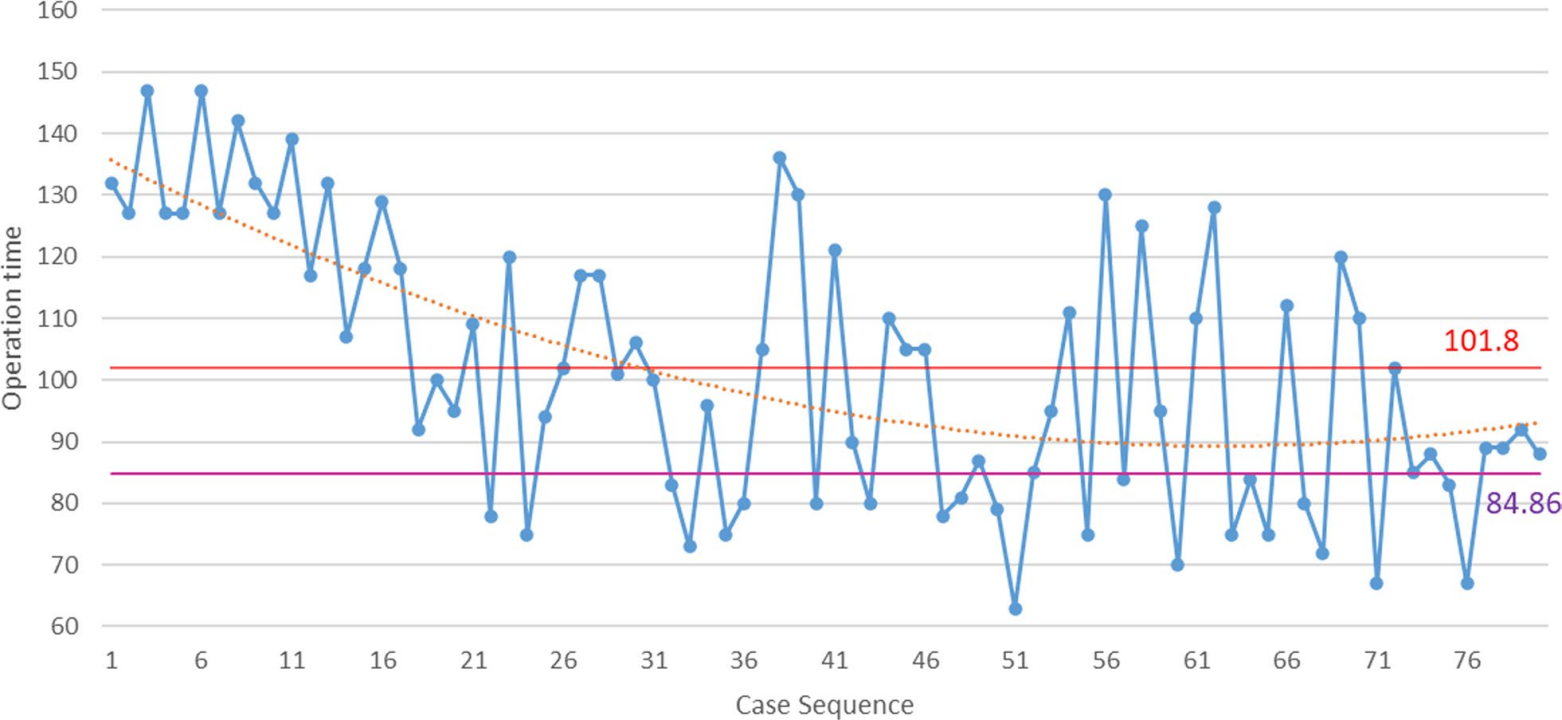
Duration of surgery for the 80 patients undergoing robot-assisted THA in chronological order, the red line representing the mean duration for the RA-THA group, the purple line representing the mean for the conventional THA group

In the present study, 80 consecutive patients underwent robot-assisted primary unilateral THA, including 42 males and 38 females. Of these, 11 were cases of congenital hip dysplasia, 42 had osteonecrosis of the femoral head, 8 had suffered femoral neck fracture, and 19 experienced primary hip arthritis. During the follow-up period, no complications were observed, including periprosthetic fracture, hip dislocation, aseptic loosening, or periprosthetic infection. For all imaging measurements, the intra-observer and inter-observer’s ICC were greater than 0.81, and the reliabilities were nearly perfect. The duration of surgery ranged from 63 to 147 min (mean: 101.8 ± 21.97 min). There was an overall downward trend in duration during the first 80 cases of RA-THA (Fig. 1).

**Fig. 2:**
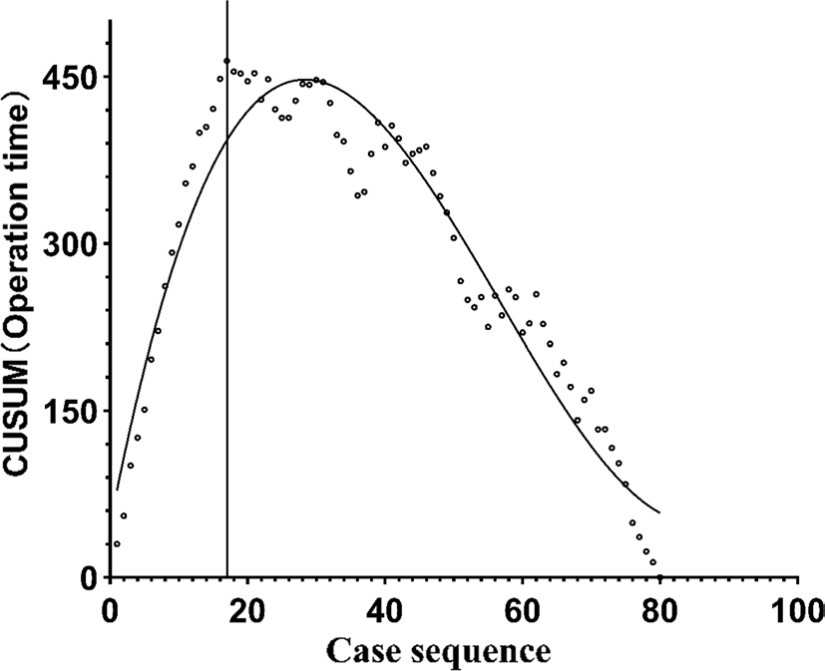
CUSUM learning curve

The learning curve was analyzed by cumulative summation. From the CUSUM plot (Fig. 2), the CUSUM peak occurred in the 17th case, representing the point at which the learning curve separated the learning period from the proficiency stage. The CUSUM learning curve was fitted to the following third-order polynomial equation: CUSUM (Duration of surgery) = 47.174 + 31.756x-0.750x^2^ + 0.004x^3^, *R*^2^ = 0.929.

The two phases were compared in terms of demographics, preoperative clinical data, duration of surgery, postoperative HHS, acetabular cup position, postoperative LLD, and postoperative offset (Table 1). There were no significant differences in preoperative age, BMI, sex, surgical side, or preoperative HSS between the two groups. The mean duration of surgery was 129.12 ± 10.71 min in the learning group and 94.43 ± 18.04 min in the proficiency group, a difference that was statistically significant (*P* < 0.001). In terms of clinical results, no significant difference in postoperative HHS score was observed between the two groups (*P* > 0.05). There was also no significant difference in the radiographic results, including postoperative acetabular cup position, postoperative LLD, or offset. For complications due to the robotic assistance, unacceptable LLD, offset, and acetabular cup position were observed during postoperative follow-up with a total incidence rate of 22.5%, and with no significant difference between the groups (*P* > 0.05). There were no robot-related complications, such as needle track infection, or peri-needle fracture in either group.

**Table 1:** Comparison of demographic data of Learning group vs. Proficiency group and Proficiency group vs. Conventional group

	Learning group	Proficiency group	*P*-value	Conventional group	*P*-value
N	17	63		80	
Age	60.06(6.65,44–69)	58.19(9.77,34–77)	0.460	56.70(10.62,28–82)	0.390
BMI, kg/m^2^	23.95(3.62,18.30–31.10)	24.40(3.22,18.40–34.30)	0.625	24.72(3.04, 17.65–31.51)	0.544
*Diagnosis *[*case* (%)]					
ONFH	9(52.9%)	33(52.4%)		45(56.3%)	
DDH	5(29.5%)	19(30.2%)		20(25.0%)	
Femoral neck fracture	3(17.6%)	11(17.4%)	0.998	15(18.7%)	0.789
*Gender *[*case *(%)]					
Male	10(58.8%)	32(50.8%)		42(52.5%)	
Female	7(41.2%)	31(49.2%)	0.556	38(47.5%)	0.839
*Surgical side *[*case *(%)]					
Left	9(52.9%)	34(54.0%)		40(50.0%)	
Right	8(47.1%)	29(46.0%)	0.940	40(50.0%)	0.637
Preoperative HHS	48.65(12.85,12–62)	53.95(14.47,15–82)	0.205	51.43(13.55,21–84)	0.359

*BMI* Body mass index, *HHS* Harris hip score; * statistically significant difference (*P* < 0.05)

**Fig. 3:**
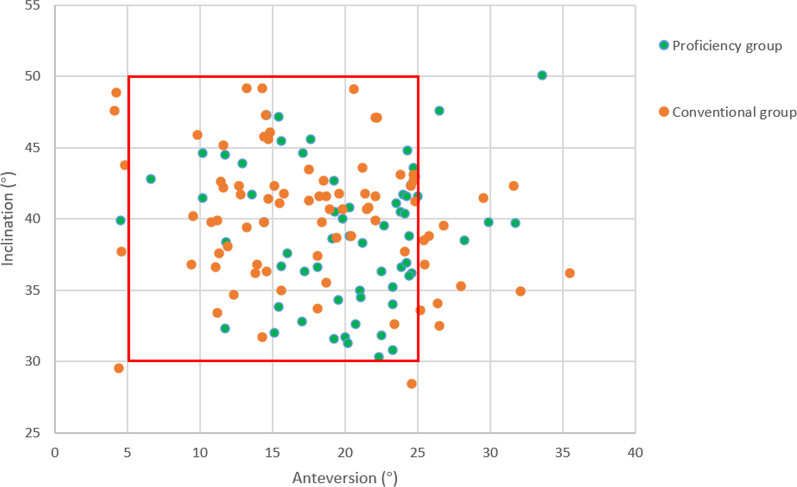
Scatter diagram of acetabular cup positioning of the proficiency robot-assisted THA and conventional THA groups

The demographics, duration of surgery, and the clinical and imaging results of RA-THA performed by the same surgeon (cases 18–80) were compared with 80 cases of conventional THA over the same period. As displayed in Table 2, there was no significant difference in patient demographics, or preoperative clinical or radiographic data between the two groups (*P* > 0.05). Additionally, there were no significant differences in acetabular inclination angle or offset between groups (*P* > 0.05). The proportion of postoperative acetabular prostheses within the Lewinnek safe zone in the proficiency or conventional groups was 90.5% and 77.5%, respectively, a difference that was statistically significant (*P* = 0.039) (Fig. 3). The postoperative acetabular anteversion was 20.17 ± 6.50° in the proficiency group and 17.84 ± 6.81° in the conventional group, differences that were statistically significant (*P* = 0.040), while postoperative LLD was 6.00 (5.00) mm in the proficiency group and 8.09 (4.33) mm in the conventional group (*P* < 0.001). The postoperative HHS of the proficiency group was 89.03 ± 7.72 while that of the conventional group was 88.76 ± 5.79, a difference that was not significant (*P* > 0.05). The duration of surgery was 94.43 ± 18.04 min in the proficiency group and 84.86 ± 14.26 min in the conventional group, which was statistically significantly different (*P* = 0.001).

**Table 2:** Comparison of data values of Learning group vs. Proficiency group and Proficiency group vs. Conventional group

	Learning group	Proficiency group	*P*-value	Conventional group	*P*-value
Duration of surgery	129.12(10.71,107–147)	94.43(18.04,63–136)	0.000*	84.86(14.26,57–129)	0.001*
Cup anteversion	20.92(7.93,8.10–39.60)	19.96(5.68,4.50–33.60)	0.573	17.84(6.81,4.10–35.50)	0.049*
Cup inclination	37.59(5.60,24.10–44.50)	38.97(4.78,30.30–50.10)	0.314	40.18(4.59,28.40–49.20)	0.127
Lewinnek safe zone (%)	14 (82.4%)	57 (90.5%)	0.347	62 (77.5%)	0.039*
LLD, mm[M(IQR)]	7.0 (3.0)	6.0 (5.0)	0.251	8.09 (4.33)	0.000*
Acceptable LLD (%)	15(88.2%)	60 (95.2%)	0.290	69(86.3%)	0.073
Offset-D, mm[M(IQR)]	3.12 (2.15)	2.83 (2.30)	0.934	3.45 (2.40)	0.067
Acceptable offset (%)	15 (88.2%)	57 (90.5%)	0.785	69(86.3%)	0.438
Postoperative HHS	92.12(7.47,77–100)	89.03(7.72,68–100)	0.145	88.76(5.79,72–100)	0.818

*LLD* Leg length discrepancy, *HHS* Harris hip score; Offset-D = offset discrepancy; *statistically significant difference (*P* < 0.05)

The absolute values of anteversion in learning group were 3.40(5.90) and in proficiency group were 4.10(3.10), and with no significant difference between the groups (*P* > 0.05). But in conventional group was 5.40(6.20) (*P* = 0.011). The absolute values of inclination in learning group were 3.70(4.10), in proficiency group were 3.60(4.50), and in conventional group were 2.70(4.25) with no significant difference between the groups (*P* > 0.05) (Table 3).

**Table 3:** Results of absolute values of the measured postoperative angles from the target angles of Learning group vs. Proficiency group and Proficiency group vs. Conventional group

	Learning group	Proficiency group	*P*-value	Conventional group	*P*-value
Cup anteversion[M(IQR)]	3.40(5.90)	4.10(3.10)	0.897	5.40(6.20)	0.011*
Cup inclination[M(IQR)]	3.70(4.10)	3.60(4.50)	0.906	2.70(4.25)	0.491

^*^ significantly different (*P* < 0.05)

Table 3 Results of absolute values of the measured postoperative angles from the target angles of Learning group vs. Proficiency group and Proficiency group vs. Conventional group.

## Discussion

Analysis of the duration of surgery using CUSUM suggests that the learning curve displayed an inflection point after 17 cases for a surgical team with extensive experience. The learning curve was divided into learning and proficiency groups at this inflection point. The mean duration of surgery was 129.12 ± 10.71 min for the learning group and 94.43 ± 18.04 min for the proficiency group, a difference that was statistically significant (*P* < 0.001). However, no statistically significant difference was found between the two groups for demographics, position of prosthesis placement, postoperative LLD, postoperative offset-D, or postoperative HHS (*P* > 0.05). The demographics, duration of surgery, postoperative radiographic results and the clinical outcomes for patients having conventional THA were compared with those receiving proficiency RA-THA, the results demonstrating that for 90.5% of cases, the position of the acetabular prosthesis postoperatively was in the Lewinnek safe zone in the proficiency group and 77.5% in the conventional group, a significant difference (*P* = 0.039). The postoperative acetabular anteversion angle was 19.96 ± 5.68° in the proficiency group and 17.84 ± 6.81° in the conventional group, a difference that was significant (*P* = 0.049), as was postoperative LLD at 6.00 (5.00) mm in the proficiency group and 8.09 (4.33) mm in the conventional group (*P* < 0.001). The absolute values of anteversion in proficiency group was 4.10(3.10) and in in conventional group was 5.40(6.20) with significant difference between the groups (*P* = 0.011). However, the duration of surgery was still longer for the proficiency group than the conventional group, a difference that was statistically significant (*P* < 0.05). These results indicate that this robot-assisted THA system is superior in terms of the control of cup anteversion and leg length.

A number of studies have evaluated the learning curves of robot-assisted THA systems [22–24]. In a previous study, Redmond et al*.* found differences in the duration of surgery and learning curves for RA-THA systems by comparing the results of three cohorts of 35 patients [25]. CUSUM is now considered a reliable method for analysis of the inflection point of a learning curve, based on the duration of surgery [26]. The research of Kong et al*.* demonstrated that the inflection point of the learning curve occurred at the 14th case for a Mako robot-assisted THA system using CUSUM and LC-CUSUM [14]. Guo et al*.* also analyzed the learning curve of the Mako RA-THA system using CUSUM, finding that the learning curve had an inflection point at the 13th case, and that the accuracy of acetabular prosthesis implantation and LLD in the proficiency RA-THA group was superior than in the conventional group [2]. Those observations are consistent with the results of the present study. Although the learning curves for RA-THA systems such as Mako and ROBODOC have been reported in the literature, to the best of our knowledge, no study of the learning curve of this novel seven-axis robot-assisted THA system has been published. Therefore, the present retrospective study was performed to analyze its learning curve and clinical effectiveness.

Dislocation is a common and unavoidable complication after THA surgery. According to the relevant literature, the incidence of dislocation after primary THA is 0.5–7% [27], [28]. Prosthesis dislocation is related to many factors, among which the inclination and anteversion of the acetabular prosthesis are important factors affecting the dislocation rate and one of the most common causes of dislocation in clinic [29]. LLD can affect the life of patients to varying degrees, significantly reducing the quality of life of patients after surgery [30]. Hip offset is related to improving range of motion, hip stability, abducting function and reducing prosthetic wear [15]. The results demonstrate that the RA-THA system significantly reduces LLD, obtains a more accurate acetabular anteversion angle, and provides a higher proportion of prosthesis positioning in the Lewinnek safe zone compared with conventional THA. Ng et al*.* analyzed the effectiveness of RA-THA systems by meta-analysis and found that 77–100% of the acetabular prostheses were located in the Lewinnek safe zone compared with only 30%-82% for conventional THA [23]. Kayani et al*.* found that a robot-assisted THA system had a short learning curve, with no significant difference in acetabular cup positioning, LLD, or offset-D across the learning curve [24]. Meanwhile, the absolute error between target angle and postoperative measured angle of anteversion was statistically significant in the proficiency group and the conventional group. This result directly reflects the accuracy of the robot-assisted system. The present research supports these results. No significant difference in LLD, acetabular prosthesis positioning, offset-D, or HHS was found throughout the learning curve. No robot-related surgical complications were observed in either of the two RA groups during the postoperative follow-up period. Additionally, there was no significant difference in the overall incidence of other complications between RA groups (*P* > 0.05), indicating that the system maintained its accuracy during the learning curve and reflected the advantages of the system. Bitar et al*.* found that the mean LLD using an RA-THA system was smaller than conventional THA, with no significant difference between the two for the percentage of procedures with an unacceptable LLD [31]. On the one hand, because the conventional group carried out preoperative planning on two-dimensional (2D) plain film, the accuracy of this method is low due to the influence of X-ray magnification, pelvic position, femoral position and projection position [32], [33]. On the other hand, the robot-assisted THA group uses the method of three-dimensional CT reconstruction for preoperative planning with high accuracy. Sariali has reported that 3D is more accurate in planning LLD than 2D [34]. It is worth noting that the plain film can only show the 2D structure of the hip, it is difficult to clearly obtain the anterior and posterior diameter of the acetabulum, and it is impossible to accurately plan the inclination angle and anteversion angle of the acetabular prosthesis. This may affect the results of the study. Furthermore, joint stability after THA is very important for patients, and combined anteversion is one of the important criteria to judge hip stability [35]. The difference in preoperative planning between the RA-THA group and the conventional group may affect the accuracy of controlling the combined anteversion. The present study also achieved similar results, demonstrating that LLD can be well controlled, even without the assistance of a robotic system, possibly due to the experience of the surgical team. Kong et al*.* found no statistically significant difference in HSS between robot-assisted and conventional THA [14], consistent with the present study. This is because improvements in acetabular positioning and leg length due to robotic-assistance will not be reflected in the clinical results over such a short period. Nevertheless, whether robot-assisted THA will ultimately provide superior functional results over the long-term compared with conventional THA remains controversial [23].

In the present study, the RA hip arthroplasty system used a 7-axis robotic arm, in theory more similar to the characteristics of movement of a unilateral arm of the human body than other systems. The robotic arm provided a large degree of movement capability without any reduction in stability. In addition, this system is compact and flexible in terms of volume, occupying only a small space in the operating room, but overcomes obstacles more easily in narrow spaces than other robot-assisted THA systems on the market because the design of the human–computer interaction strategy, which was optimized for the needs and surgical habits of Chinese doctors. Although the operational performance of the RA system is excellent and the functional outcomes of patients are stable, there were 6 patients in the proficiency group whose acetabular prostheses were not within the Lewinnek safe zone. Of these, 5 patients had lumbar disk herniation which caused pelvic retroversion in the standing position, requiring anteversion of the acetabular prosthesis of more than 25° in order to maintain stability of the hip joint during surgery. One patient had developmental dysplasia of the hip causing the femoral anteversion angle to increase [36]. To maintain the combined anteversion angle within an appropriate range, the acetabular anteversion angle was inevitably reduced during surgery resulting in an acetabular prosthesis anteversion angle of less than 5° [37]. The results of the present study demonstrate that postoperative prosthesis positioning and LLD of the proficiency group using the novel RA system are superior to conventional THA. However, there are differences in acetabular prosthesis positioning, LLD, and offset compared with other RA-THA systems that require further exploration.

The present study inevitably had a number of limitations. As a retrospective study, there were multiple biases, including selection bias, evaluation bias, and measurement bias, which were difficult to avoid. Additionally, the sample size was small and only a few parameters were observed, both of which should be increased in future studies to verify the effectiveness and ultimate learning curve of this robotic system. Furthermore, as a single-center study, the learning curve we obtained may not apply to other clinical centers.

## Conclusions

In summary, the learning curve for a novel seven-axis robot-assisted THA system was evaluated. In terms of the duration of surgery, there was a learning curve of 17 cases. For the other parameters measured, no learning curve was observed, in terms of leg length discrepancy, hip offset, or the accuracy of acetabular prosthesis positioning. During the proficiency phase, the RA system was found to be superior to conventional methods in controlling leg length and the accuracy of acetabular cup placement.

## Data Availability

The data are not publicly available, as participants in this study need their data for further analysis.

## Abbreviations

RA-THA: Robot-assisted total hip arthroplasty
LLD: Leg length discrepancy
CUSUM: Cumulative sum
HHS: Harris hip score
BMI: Body mass index
